# SwissBioPics—an interactive library of cell images for the visualization of subcellular location data

**DOI:** 10.1093/database/baac026

**Published:** 2022-04-12

**Authors:** Philippe Le Mercier, Jerven Bolleman, Edouard de Castro, Elisabeth Gasteiger, Parit Bansal, Andrea H Auchincloss, Emmanuel Boutet, Lionel Breuza, Cristina Casals-Casas, Anne Estreicher, Marc Feuermann, Damien Lieberherr, Catherine Rivoire, Ivo Pedruzzi, Nicole Redaschi, Alan Bridge

**Affiliations:** Swiss-Prot group, SIB Swiss Institute of Bioinformatics, CMU - 1 rue Michel Servet CH-1211 Geneva 4, Switzerland; Swiss-Prot group, SIB Swiss Institute of Bioinformatics, CMU - 1 rue Michel Servet CH-1211 Geneva 4, Switzerland; Swiss-Prot group, SIB Swiss Institute of Bioinformatics, CMU - 1 rue Michel Servet CH-1211 Geneva 4, Switzerland; Swiss-Prot group, SIB Swiss Institute of Bioinformatics, CMU - 1 rue Michel Servet CH-1211 Geneva 4, Switzerland; Swiss-Prot group, SIB Swiss Institute of Bioinformatics, CMU - 1 rue Michel Servet CH-1211 Geneva 4, Switzerland; Swiss-Prot group, SIB Swiss Institute of Bioinformatics, CMU - 1 rue Michel Servet CH-1211 Geneva 4, Switzerland; Swiss-Prot group, SIB Swiss Institute of Bioinformatics, CMU - 1 rue Michel Servet CH-1211 Geneva 4, Switzerland; Swiss-Prot group, SIB Swiss Institute of Bioinformatics, CMU - 1 rue Michel Servet CH-1211 Geneva 4, Switzerland; Swiss-Prot group, SIB Swiss Institute of Bioinformatics, CMU - 1 rue Michel Servet CH-1211 Geneva 4, Switzerland; Swiss-Prot group, SIB Swiss Institute of Bioinformatics, CMU - 1 rue Michel Servet CH-1211 Geneva 4, Switzerland; Swiss-Prot group, SIB Swiss Institute of Bioinformatics, CMU - 1 rue Michel Servet CH-1211 Geneva 4, Switzerland; Swiss-Prot group, SIB Swiss Institute of Bioinformatics, CMU - 1 rue Michel Servet CH-1211 Geneva 4, Switzerland; Swiss-Prot group, SIB Swiss Institute of Bioinformatics, CMU - 1 rue Michel Servet CH-1211 Geneva 4, Switzerland; Swiss-Prot group, SIB Swiss Institute of Bioinformatics, CMU - 1 rue Michel Servet CH-1211 Geneva 4, Switzerland; Swiss-Prot group, SIB Swiss Institute of Bioinformatics, CMU - 1 rue Michel Servet CH-1211 Geneva 4, Switzerland; Swiss-Prot group, SIB Swiss Institute of Bioinformatics, CMU - 1 rue Michel Servet CH-1211 Geneva 4, Switzerland

## Abstract

SwissBioPics (www.swissbiopics.org) is a freely available resource of interactive, high-resolution cell images designed for the visualization of subcellular location data. SwissBioPics provides images describing cell types from all kingdoms of life—from the specialized muscle, neuronal and epithelial cells of animals, to the rods, cocci, clubs and spirals of prokaryotes. All cell images in SwissBioPics are drawn in Scalable Vector Graphics (SVG), with each subcellular location tagged with a unique identifier from the controlled vocabulary of subcellular locations and organelles of UniProt (https://www.uniprot.org/locations/). Users can search and explore SwissBioPics cell images through our website, which provides a platform for users to learn more about how cells are organized. A web component allows developers to embed SwissBioPics images in their own websites, using the associated JavaScript and a styling template, and to highlight subcellular locations and organelles by simply providing the web component with the appropriate identifier(s) from the UniProt-controlled vocabulary or the ‘Cellular Component’ branch of the Gene Ontology (www.geneontology.org), as well as an organism identifier from the National Center for Biotechnology Information taxonomy (https://www.ncbi.nlm.nih.gov/taxonomy). The UniProt website now uses SwissBioPics to visualize the subcellular locations and organelles where proteins function. SwissBioPics is freely available for anyone to use under a Creative Commons Attribution 4.0 International (CC BY 4.0) license.

**Database URL:**

www.swissbiopics.org

## Introduction

All living organisms from whales to bacteria are made up of the same basic building blocks, first referred to as ‘cells’ by Robert Hooke in his landmark publication in 1665, *Micrographia: or some physiological definitions of minute bodies* (1). Cells come in a bewildering array of shapes and sizes; there are hundreds of morphologically distinct cell types in the human body alone (2), although the true number of functionally distinct cell types at the molecular level will likely be much greater (3).

Cells themselves are composed of distinct subcellular structures, commonly referred to as organelles. Organelles provide structure and organization to the cell; they carry out a broad range of functions and divide the cellular machinery into distinct regulatory and metabolic regions. The first organelle to be named in a scientific publication, by Robert Brown in 1833, was the cell nucleus, which of course characterizes eukaryotes; since that time, hundreds of more organelles have been characterized from both eukaryotes and prokaryotes, including organelles such as the magnetosome, which allows bacteria to navigate according to the magnetic poles of our Earth (4). The evolutionary history of organelles is as fascinating as their functional and morphological diversity: Mitochondria and chloroplasts evolved from a process of endosymbiosis, endowing cells with new capabilities such as aerobic metabolism and photosynthesis (5, 6).

Understanding the spatial organization of cells and their building blocks is essential to understanding life. Knowledge of the subcellular localization of proteins is provided by knowledge resources such as the Universal Protein Resource (UniProt, www.uniprot.org) (7) and model organism databases (MODs) such as Saccharomyces Genome Database (8), PomBase (9), WormBase (10), FlyBase (11), Mouse Genome Informatics (12) and Rat Genome Database (13). Expert curators from these resources capture knowledge of protein subcellular locations from the scientific literature in forms that are FAIR (14) using controlled vocabularies and ontologies, including the UniProt-controlled vocabulary for subcellular locations (https://www.uniprot.org/locations/) and the Gene Ontology (GO) ‘Cellular Component’ branch, which is also used by UniProt as well as the MODs and other resources (www.geneontology.org) (15). These resources also integrate other sources of information on subcellular locations, including high-throughput proteomics and microscopy studies (such as (16)) and computational predictions (17, 18). Other notable resources of subcellular location data include COMPARTMENTS (19), which integrates and scores knowledge from the aforementioned resources and others such as the Human Protein Atlas (20) to provide a one-stop shop for subcellular location data.

GO and UniProt annotations are normally presented to users in the form of lists of annotated terms, which provide excellent support for computational biology, but which fail to highlight for the novice human reader how organelles such as the nucleus and nucleolus, the early and late Golgi, or the microtubule and centrosome are related. The most natural and effective way to present these annotations and many other types of biological data is probably in visual form (21). Visualizations allow biologists to grasp relationships and patterns that are not obvious from text rapidly. The COMPARTMENTS database for subcellular location provides images of cells on which users can map subcellular location data. These images were used in UniProt for many years, but they cover only a small fraction of the hundreds of annotated subcellular locations in UniProt. Reactome also provides a library of attractive biological icons for pathway diagrams, which covers proteins, chemicals, organelles and tissues and focuses on human biology (22). In this work, we describe the creation of a resource of high-quality cellular images for the visualization and interpretation of subcellular location annotations that will eventually grow to cover all annotated subcellular locations and taxonomic groups represented in UniProt. This resource is ‘SwissBioPics’. A biographical picture or ‘biopic’ is a film that tells the story of a life; SwissBioPics tells the stories of many cellular lives or varieties of life in graphical form.

## Results

### SwissBioPics cell image library

**Figure 1.**
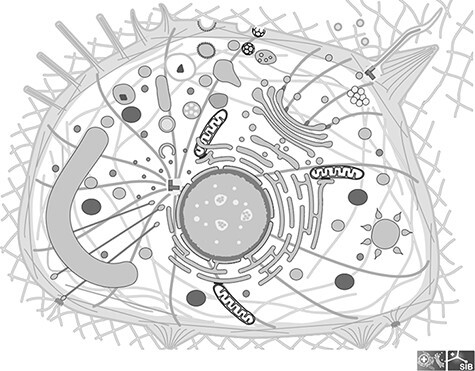
Image of a generic cell from the Clade Eumetazoa; This cell illustrates all the cellular components found in any Eumetazoan, although in reality no cell carries all of them; All components are individual groups in the SVG file, which can be hidden or colored.

The image library of SwissBioPics, available for download at www.swissbiopics.org, currently includes images for 48 different cell types from all kingdoms of life. There are 24 types of bacterial and archaeal cells, including cells with single or double membranes (Gram+ and Gram−) and covering morphologies as diverse as cocci, rods, spirals, commas and clubs. Fungal cells include a generic fungal hypha, as well as budding and fission yeasts, providing good coverage of the most common fungal model organisms. Eukaryotic cells include a generic eukaryotic cell, a plant cell, a unicellular algal cell, an apicomplexan cell, a cnidocyte and a series of specialized animal cells—epithelial, neuronal, muscle, photoreceptor, egg and spermatozoa. Within each cell, the organelles have a realistic morphology, designed based on extensive research of literature and microscopy data, although their relative sizes are not necessarily accurate. We adjusted these to make sure that each organelle was clearly visible to the viewer of the cell image on first viewing, without needing to zoom in. Figure 1 shows an image of a generic cell from the Clade Eumetazoa. Individual species within the taxonomic range covered by a cell image may vary in the morphology of certain organelles, for instance some species of budding yeasts have a stacked Golgi and others a dispersed Golgi. For such cases, we choose the morphology that is more widely recognized by nonexpert users.

Each cell image is mapped to one or more identifiers from the curated National Center for Biotechnology Information (NCBI) taxonomy (https://www.ncbi.nlm.nih.gov/taxonomy) to specify the correct taxonomic grouping for the cell type; their number and rank may vary according to the taxonomic distribution of the morphology described by the cell image in question. For instance, generic images for ‘animal’ and ‘plant’ cells are mapped to a single identifier for the appropriate clade (Eumetazoa and Viridiplantae, respectively), while the image for ‘rod shaped bacterial cells with a single membrane’ is mapped to the taxonomic identifier of each valid genus (a list currently including *Bacillus, Clostridium, Lactobacillus, Listeria* and *Geobacillus*, to name but a few). We provide a complete mapping of cell images to identifiers from the NCBI taxonomy in the Supplementary Material.

Within the Scalable Vector Graphics (SVG) file of an image, each organelle or subcellular location is tagged with an identifier from the UniProt-controlled vocabulary of subcellular location terms and the name and description of each term are included as metadata. Each image covers a range of species, and some of those species may have specialized organelles not found in others. For example, some unicellular eukaryotes have a chloroplast, but most do not. We hide these specialized or restricted organelles by default in the SVG file; the SwissBioPics web component (see the following section) will reveal hidden organelles when receiving a request to highlight them.

### SwissBioPics website

**Figure 2.**
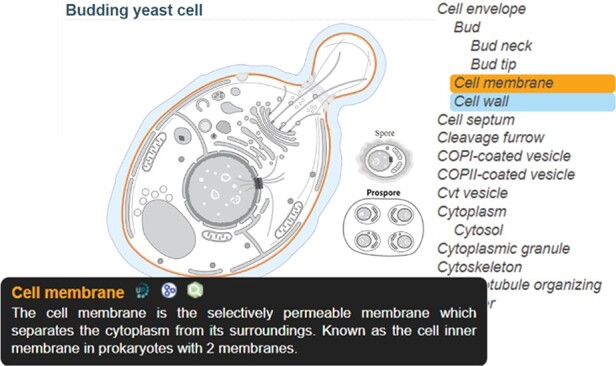
SwissBioPics page for budding yeast cell; The ‘Cell membrane’, selected on the sidebar list, is colored orange, with a tooltip showing a description of the organelle and links to the UniProt, AmiGO and QuickGO websites; The mouse is hovering on ‘Cell wall’ in the list, coloring this part blue.

The SwissBioPics website www.swissbiopics.org allows users to browse, explore and download the image library. A single page presents each image along with a sidebar that lists the subcellular locations or organelles available for it (Figure 2). Users can zoom in and out of the image using a mouse wheel and select subcellular locations or organelles either from the image itself or from the sidebar list to the right. Hovering over a location highlights the name and drawing (in blue); clicking on a location highlights the name and drawing (in orange) and displays an information tooltip. This tooltip provides a description of the subcellular location or organelle selected (taken from UniProt), as well as links to UniProt, AmiGO and QuickGO. Users can download the SVG of the image by clicking on an arrow to the left of the image, but to embed images in their own websites and dynamically highlight subcellular locations or organelles we recommend using the SwissBioPics web component which selects the taxonomically correct image and, if required, reveals hidden organelles and maps GO identifiers to UniProt subcellular location identifiers.

### SwissBioPics web component

**Figure 3.**
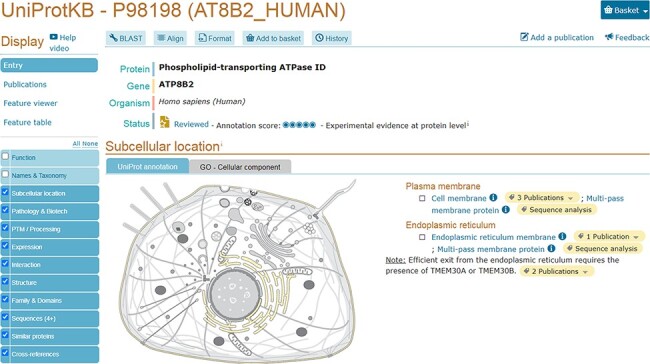
UniProtKB entry AT8B2_ HUMAN (P98198) shows an embedded SwissBioPics image: The generic animal cell (Eumetazoa) is selected based on organism taxonomy; the cell membrane and endoplasmic reticulum are highlighted using annotations from the UniProt entry.

**Figure 4.**
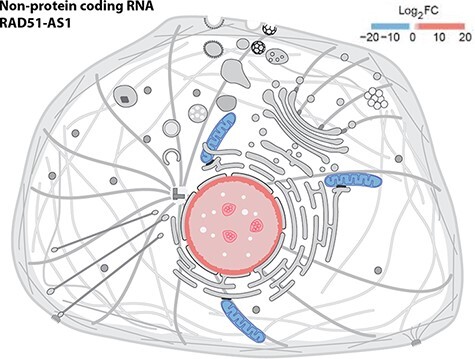
Displaying quantitative data in SwissBioPics using colors. The image shows the fold change (FC) of the RAD51-AS1 non-protein-coding transcript in the nucleus and mitochondrion (26).

We provide a reusable web component that allows developers to use SwissBioPics images in their own websites to visualize annotations of subcellular locations and organelles. The code and technical documentation required to do this are available at https://www.npmjs.com/package/@swissprot/swissbiopics-visualizer. The web component requires at least two parameters: the identifier for the organism, from the NCBI taxonomy, and the identifier(s) for the subcellular locations or organelles, from the UniProt-controlled vocabulary or the GO ‘Cellular Component’ branch. Given this information, the web component uses an application programming interface (API) to automatically select the most appropriate image that maximizes the number of displayed annotations and to highlight these locations on the image. Developers can override the default styling of subcellular locations using Cascading Style Sheets (CSS) rules (the CSS styling derives from the identifier for the subcellular location of UniProt or GO ‘Cellular Component’) to assign for instance different colors to annotations supported by different evidence codes, as in the UniProt website, which now uses SwissBioPics to display the subcellular locations and organelles where proteins function (Figure 3), or to display quantitative data as a heat map, using different colors for different value ranges (Figure 4).

## Discussion

SwissBioPics provides a freely available library of cell images, website and web component for the visualization of subcellular location data. The images are realistic in terms of morphology (although not necessarily scale) and resemble a combination of electron micrographs and textbook drawings. The website provides a means to search and browse the image library and to learn about the morphology of cells, while the web component allows developers to embed SwissBioPics in their own websites in order to display subcellular location data themselves.

Although originally designed to visualize protein location(s) in UniProt, SwissBioPics can be used to visualize the location(s) of any biological entity for which such annotations are available, including molecules such as RNAs (23) or small molecule metabolites (24) or complexes (25). All that is required is an NCBI taxonomy identifier and a list of subcellular location(s) expressed using stable unique identifiers from the UniProt-controlled vocabulary for subcellular locations or the ‘Cellular Component’ branch of the GO.

The SwissBioPics image library currently covers around 72% of all subcellular locations described in UniProtKB and 95% of all entries in UniProtKB/Swiss-Prot for which a subcellular location annotation is available. We are currently designing new images that will expand the coverage of SwissBioPics images—our goal is to cover all UniProtKB subcellular locations eventually. We invite readers and potential users of SwissBioPics to contact us at https://www.swissbiopics.org/ with suggestions for new images describing cells and subcellular locations and organelles of particular interest to them.

## Materials and methods

### Image generation

We draw cell images using Adobe illustrator in grayscale. Each subcellular location is a distinct layer in the image and is assigned a tag of the type <SLXXXX>, in which SLXXXX is the identifier for the corresponding term from the UniProt-controlled vocabulary for subcellular locations (for example ‘SL0041’ for ‘Cell wall’). Layers are disposed in a hierarchy corresponding to that of the UniProt-controlled vocabulary for subcellular locations. Each picture is ‘saved as’ an SVG file comprising only the graphics, using the SVG1.1 Illustrator profile.

### Web component and API

The web component talks to an API that decides which image to display based on information provided by the developer invoking the web component—namely an NCBI taxonomy identifier and identifier(s) from the UniProt-controlled vocabulary for subcellular locations or the GO ‘Cellular Component’ branch. The API enriches the original SVG files by adding the name and description of each UniProt subcellular location term. When the API is called with GO identifiers, it maps them to the corresponding identifiers from the UniProt-controlled vocabulary to tag these subcellular locations in the image with the GO identifiers for custom CSS styling. The returned image is then available for further styling and display. The API is implemented as a small Java-based Vert.x webserver. It uses the UniProt SPARQL endpoint (https://sparql.uniprot.org/sparql) to retrieve the full NCBI taxonomy data and the UniProt-controlled vocabulary for subcellular locations with its mapping to the GO ‘Cellular Component’ branch.

### Website

The website www.swissbiopics.org is served by a custom engine (written in Scala) running under the Java Virtual Machine on Linux servers. It consists of a card view of available cell images that each link to individual cell pages. Cell pages have dynamic highlighting of location information and zoom handled by custom JavaScript independent of the web component. The site also provides a search interface, including auto-generated search suggestions for location names and cell names, using Apache Lucene.

## Supplementary Material

baac026_Supp

## Data Availability

SwissBioPics is available under a Creative Commons Attribution 4.0 International (CC BY 4.0) License.
